# NRI and SIRI are the optimal combinations for prognostic risk stratification in patients with non-small cell lung cancer after EGFR-TKI therapy

**DOI:** 10.1007/s12094-024-03735-7

**Published:** 2024-09-20

**Authors:** Xia Liu, Peipei Wang, Guolong Liu

**Affiliations:** 1 The First Affiliated Hospital, Jinan University, Guangzhou, Guangdong China; 2 Department of Oncology, Guangzhou First People’s Hospital, South China University of Technology, Guangzhou, Guangdong China; 3 School of Biomedical Sciences and Engineering, Guangzhou International Campus, South China University of Technology, Guangzhou, Guangdong China

**Keywords:** Non-small cell lung cancer, EGFR-TKI, Risk stratification, Biomarker, NRI, SIRI

## Abstract

**Background:**

Epidermal growth factor receptor (EGFR) tyrosine kinase inhibitors (TKIs) have become the standard treatment for advanced non-small cell lung cancer (NSCLC) with EGFR mutations. However, NSCLC heterogeneity leads to differences in efficacy; thus, potential biomarkers need to be explored to predict the prognosis of patients. Recently, the prognostic importance of pre-treatment malnutrition and systemic inflammatory response in cancer patients has received increasing attention.

**Methods:**

In this study, clinical information from 363 NSCLC patients receiving EGFR-TKI treatment at our clinical center was used for analysis.

**Results:**

High nutritional risk index (NRI) and systemic inflammation response index (SIRI) were significantly associated with poor overall survival (OS) and progression-free survival (PFS) in NSCLC patients (*P* < 0.05). Importantly, NRI and SIRI were the best combination models for predicting clinical outcomes of NSCLC patients and independent OS and PFS predictors. Moreover, a nomogram model was constructed by combining NRI/SIRI, sex, smoking history, *EGFR* mutation, TNM stage, and surgery treatment to visually and personally predict the 1-, 2-, 3-, 4-, and 5-year OS of patients with NSCLC. Notably, risk stratification based on the nomogram model was better than that based on the TNM stage.

**Conclusion:**

NRI and SIRI were the best combination models for predicting clinical outcomes of NSCLC patients receiving EGFR-TKI treatment, which may be a novel biomarker for supplement risk stratification in NSCLC patients.

**Supplementary Information:**

The online version contains supplementary material available at 10.1007/s12094-024-03735-7.

## Introduction

Lung cancer remains one of the most common and invasive malignant tumors worldwide and is also the leading cause of cancer-related deaths [1]. Non-small cell lung cancer (NSCLC) accounts for 85% of all lung cancers [2]. In recent years, epidermal growth factor receptor (EGFR) tyrosine kinase inhibitors (TKIs) have become the standard treatment for advanced NSCLC with EGFR mutations [3, 4]. The first-generation EGFR-TKI gefitinib significantly provided disease-free survival (DFS) for resectable EGFR mutant NSCLC [5]. Moreover, in the ADAURA trial, positive outcomes of third-generation EGFR-TKI osimertinib were reported in patients with EGFR mutations, and osimertinib as an adjuvant therapy for patients with *EGFR exon 19 deletion* or *exon 21 L858R* mutation has been approved by the US Food and Drug Administration (FDA) [6, 7]. Although EGFR-TKI has been successful in EGFR-mutated NSCLC, its heterogeneity leads to differences in efficacy; therefore, potential biomarkers need to be explored to predict the prognosis of patients [7].

The systemic inflammatory response caused by neutrophils, lymphocytes, macrophages, and mast cells serves a critical role in tumor development, aggravating local immune suppression, and leading to cancer progression and poor clinical outcomes [8]. Therefore, the prognostic importance of inflammation-based indexes such as systemic inflammation response index (SIRI), advanced lung cancer inflammation index (ALI), lymphocyte-to-monocyte ratio (LMR), neutrophil-to-lymphocyte ratio (NLR), platelet-to-lymphocyte ratio (PLR), prognostic nutritional index (PNI), and Systemic immune-inflammation index (SII) in NSCLC has been increasingly studied [8–10]. Furthermore, malnutrition in cancer patients is associated with treatment-related toxicity, poor quality of life, decreased function, and poor prognosis [11]. Identifying patients with malnutrition can help with targeted interventions and may improve the outcomes of NSCLC patients [11]. Several different nutritional indexes have been developed to assess the prognosis of cancer patients, such as nutritional risk index (NRI) and controlling nutritional status (CONUT) [11–13]. However, it is currently unclear which tool can provide the best prognostic assessment for NSCLC patients receiving EGFR-TKI treatment.

In this study, clinical information from 363 NSCLC patients receiving EGFR-TKI treatment at our clinical center was used for analysis to evaluate nutritional and systemic inflammation-related indexes (including NRI, SIRI, ALI, CONUT, LMR, NLR, PLR, PNI, and SII), as well as their optimal combination for predicting clinical outcomes in patients with NSCLC.

## Materials and methods

### NSCLC patients

The clinical information of 363 NSCLC patients receiving EGFR-TKI treatment (primarily involving gefitinib, erlotinib, and ecetinib) was collected from Guangzhou First People’s Hospital between December 2008 and April 2019, which is listed in Table S1. The median follow-up time was 32.8 months (range: 0.6–102.4 months). Overall survival (OS) is defined as the time from the application of EGFR-TKI to death from any cause or the end of follow-up. Progression-free survival (PFS) is defined as the time from the application of EGFR-TKI to disease progression, relapse, death, or the end of follow-up. ALI was calculated as body mass index (BMI) × serum albumin (ALB)/NLR [14]. LMR = lymphocyte-to-monocyte ratio. PLR = platelet-to-lymphocyte ratio. The calculation methods for CONUT and NRI were described in previous publications [12, 13, 15]. PNI was calculated as ALB level (g/L) + 5 × total lymphocyte count (10^9^/L). SII was calculated as the platelet count × neutrophil count/lymphocyte count. SIRI was calculated as monocyte count x neutrophil count /lymphocyte count.

### Nomogram model

The nomogram model is usually used for personalized and visual prediction of clinical outcomes in cancer patients [16]. R language (version 4.3.2) was used to construct a nomogram model to visualize the 1-, 2-, 3-, 4-, and 5-year OS rates of NSCLC patients treated with EGFR-TKI. Firstly, the nomogram model assigns a point to each variable. Then, the total points of all variables for each patient were added up and located on the total point table. Finally, the OS rates were determined by drawing a vertical line on the total point scale. The calibration curves and time-dependent receiver operating characteristic (ROC) curves were used to evaluate the predictive performance of the nomogram model. The criteria for the good prognosis prediction performance of the nomogram model are as follows: (i) The predicted OS in the nomogram model is significantly close to the actual OS in the calibration curves. (ii) The area under the curve (AUC) > 0.5.

### The optimal cut-points

The optimal cut-points were obtained by the “maxstat” package or X-tile software (version 3.6.1), as appropriate [17]. This is an outcome-oriented method that identifies a cut-point that best separates survival outcomes, which are currently widely used to analyze the prognosis of cancer patients. The optimal cut-points were at the maximum x^2^ and the minimum *P* value in quantitative variables. According to the optimal cut-points, patients were divided into two or three subgroups, which were submitted to plot the Kaplan–Meier curves.

### The optimal combination model

The package "glmulti" finds what are the n best models (the confidence set of models) among all possible models using automated model selection and multimodel inference with (G) LMs [18]. Models are fitted with the “glm” fitting function and are ranked with the “aicc Information Criterion.” The best models are found using a genetic algorithm, which allows very large candidate sets to be addressed. The output can be used for model selection.

### Statistical analysis

Statistical analyses were performed using SPSS 22.0 (IBM, Armonk, New York, USA) and R (version 4.3.2, https://www.r-project.org/), as appropriate. Kaplan–Meier curves were compared using the log-rank test in the R package "survival." Differences between the two and three subgroups of quantitative data were compared by Mann–Whitney–Wilcoxon and Kruskal–Wallis tests, respectively. Univariate and multivariate Cox proportional risk regression models were used to identify independent OS and PFS factors. The Spearman method was used to evaluate the correlation between two subgroups of quantitative data. The AUC in time-dependent ROC was obtained using the package “survivalROC.” Two ROC curves were compared using the package "pROC." A two-tailed *P* < 0.05 was considered statistically significant.

## Results

### A weighted combination of NRI and SIRI predicted the prognosis of NSCLC patients treated with EGFR-TKI

**Fig. 1:**
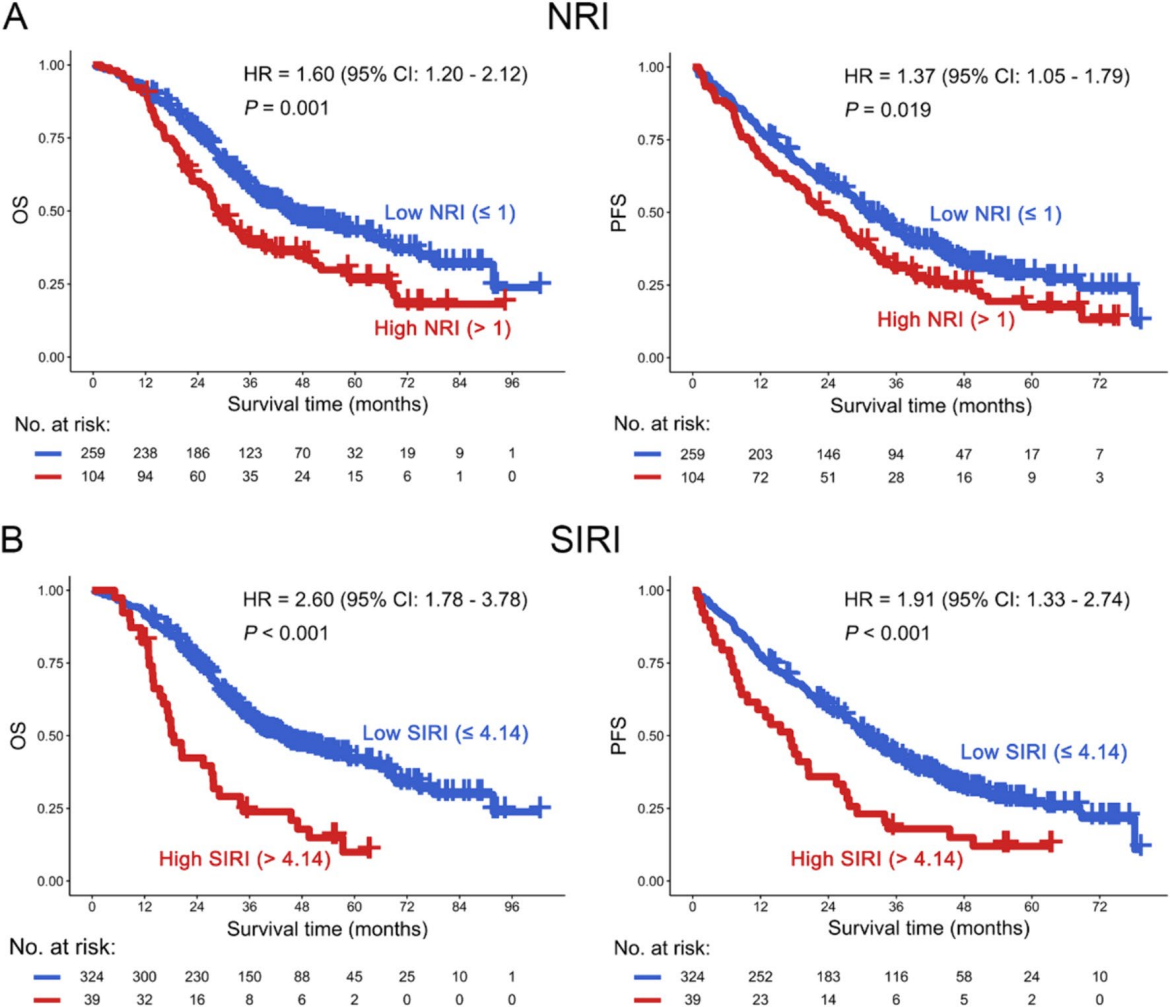
Overall survival (OS) (left panel) and progression-free survival (PFS) (right panel) analysis of NRI (**A**) and SIRI (**B**) in NSCLC patients following EGFR-TKI. NRI: Nutrition risk index; SIRI: Systemic inflammatory response index; EGFR: Epidermal growth factor receptor; TKI: Tyrosine kinase inhibitor

Kaplan–Meier curve was usually used to evaluate the quantitative or qualitative variables with patients’ outcomes [19]. Therefore, prognostic indices were analyzed using Kaplan–Meier curves. The results indicated that NSCLC patients treated with EGFR-TKI were divided into two subgroups based on the optimal cut-point, and high NRI was associated with poor OS of patients [hazard ratio (HR) = 1.60, 95% confidence interval (CI) 1.20–2.12, *P* = 0.001]. These results were confirmed in PFS (HR = 1.37, 95% CI 1.05–1.79, *P* = 0.019) (Fig. 1A and S1). Moreover, high SIRI was associated with poor OS of patients with NSCLC (HR = 2.60, 95% CI 1.78–3.78, *P* < 0.001). These results were again confirmed in PFS (HR = 1.91, 95% CI 1.33–2.74, *P* < 0.001) (Fig. 1B and S1). Additionally, low ALI, LMR, and PNI, and high CONUT, NLR, PLR, and SII predicted poor OS and PFS in patients with NSCLC (*P* < 0.05, Figure S2 and S3A–G). Moreover, the ROC curves were used to evaluate the predictive prognostic power of different indexes, and the results suggested that NRI, SIRI, ALI, LMR, NLR, PNI, and SII could significantly predict OS in NSCLC patients receiving EGFR-TKI therapy (*P* < 0.05), other than CONUT and PLR (*P* > 0.05) (Figure S4A–I). However, only ALI and LMR could predict PFS in NSCLC patients, while NRI, SIRI, CONUT, NLR, PLR, PNI, and SII were not significantly correlated with PFS in patients (*P* > 0.05) (Figure S5A–I). In addition, NRI, SIRI, ALI, CONUT, LMR, NLR, PNI, and SII could predict the response of NSCLC patients to EGFR-TKI (*P* < 0.05), except for PLR (*P* > 0.05) (Figure S6A–I).

**Fig. 2:**
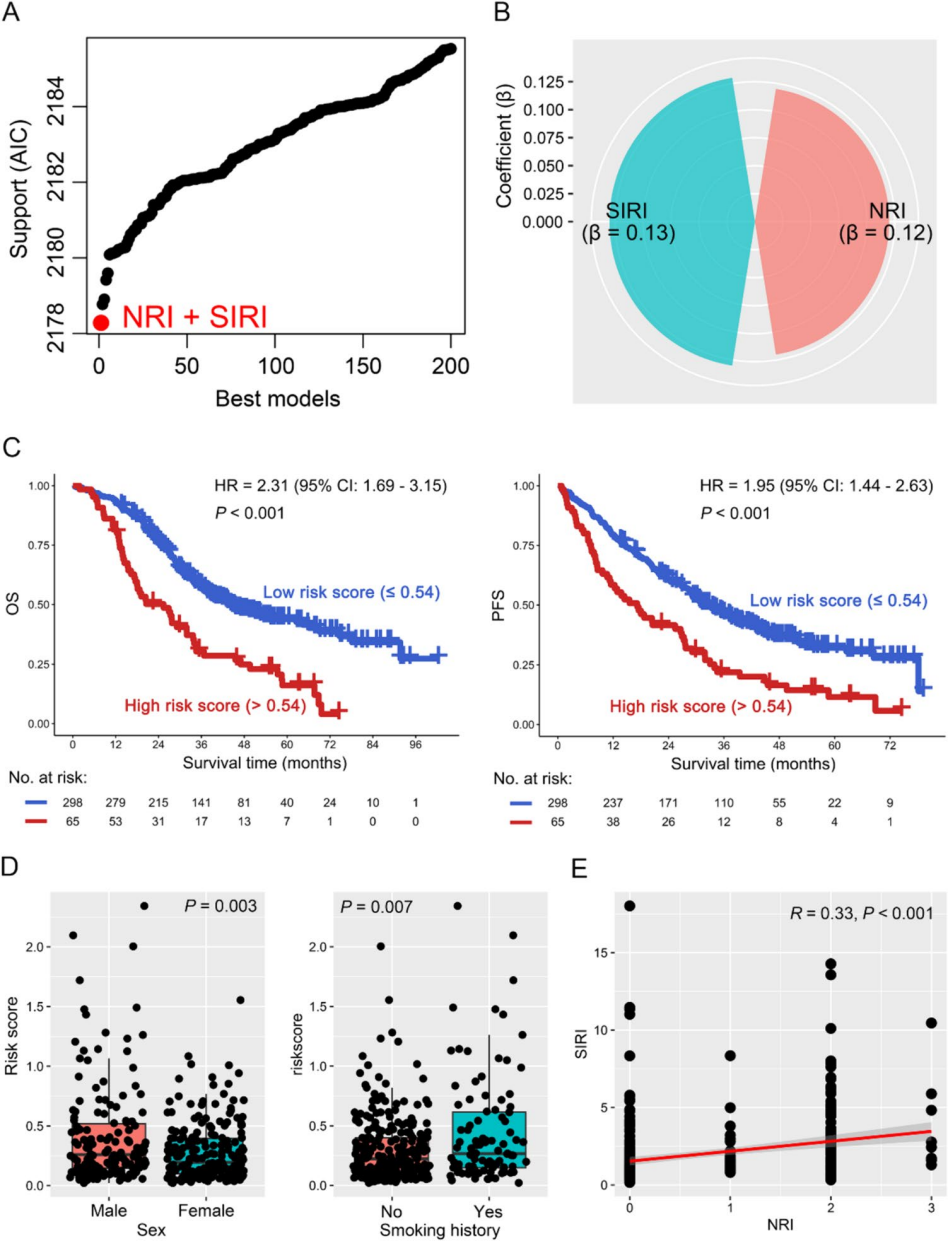
A weighted combination of NRI and SIRI was associated with the prognosis of patients with NSCLC. **A** Akaike information criterion (AIC) profile of the best to the worst model, which was determined by the package "glmulti" in R. The red dot represents the best model. **B** The contribution of NRI and SIRI to OS, which was obtained from the coefficients β in the multivariate Cox proportional risk regression model. Risk score = β1* (NRI) + β2* (SIRI). **C** OS (left panel) and PFS (right panel) analysis of risk score. **D** The relationship between risk score and sex (left panel) and smoking history (right panel). **E** The correlation between NRI and SIRI

The combination of indexes often predicts the prognosis of patients better than a single one; thus, the best combination model among the above indexes was investigated. Interestingly, a combination of NRI and SIRI was the best model for predicting clinical outcomes in NSCLC patients treated with EGFR-TKI (Fig. 2A). Then, the contributions of NRI and SIRI to OS were obtained from multivariate Cox regression analysis, and the risk score was calculated according to the following formula: risk score = 0.12 * NRI + 0.13 * SIRI (Fig. 2B). Notably, high-risk score was significantly associated with poor OS in NSCLC patients (HR = 2.31, 95% CI 1.69–3.15, *P* < 0.001), which was confirmed in PFS (HR = 1.95, 95% CI 1.44–2.63, *P* < 0.001) (Fig. 2C). When risk score, age, sex, smoking history, *EGFR* mutation, TNM stage, brain metastasis, pathology, TKI, and surgery treatment were included into univariate and multivariate Cox regression analysis to balance the impact of clinical factors, risk score was an independent OS (HR = 2.27, 95% CI 1.65–3.13, *P* < 0.001) and PFS (HR = 1.86, 95% CI 1.37–2.52, *P* < 0.001) factor in NSCLC patients receiving EGFR-TKI treatment (Table 1). The ROC curves were used to evaluate the predictive prognostic power of risk score, and the results indicated that risk score could simultaneously predict OS, PFS, and EGFR-TKI response in NSCLC patients, which was consistent with the results of the survival curves (*P* < 0.05; Figure S4J, S5J, and S6J). Furthermore, the risk score was positively correlated with male and smoking history (*P* < 0.01), other than age, TNM stage, brain metastasis, *EGFR* mutation, and pathology subtypes (*P* > 0.05) (Fig. 2D and S7A–E). Finally, as the risk score was calculated by NRI and SIRI, their relationship was investigated, and the result suggested that NRI had a positive correlation with SIRI (R = 0.33, *P* < 0.001; Fig. 2E). Accordingly, the above results suggest that a weighted combination of NRI and SIRI was the best model to predict the prognosis of NSCLC patients treated with EGFR-TKI.

**Table 1:** Univariate and multivariate regression analyses in patients with advanced lung cancer

Variables	OS				PFS			
	Univariate COX		Multivariate COX		Univariate COX		Multivariate COX	
	HR (95% CI)	*P* value	HR (95% CI)	*P* value	HR (95% CI)	*P* value	HR (95% CI)	*P* value
Risk score (ref: Low)								
High	2.31 (1.69, 3.15)	< 0.001	2.27 (1.65, 3.13)	< 0.001	1.95 (1.44, 2.63)	< 0.001	1.86 (1.37, 2.52)	< 0.001
Age, years (ref: < 60)								
≥ 60	1.09 (0.83, 1.44)	0.524			1.05 (0.82, 1.36)	0.684		
Sex (ref: Male)								
Female	0.70 (0.53, 0.92)	0.011	0.83 (0.57, 1.22)	0.342	0.81 (0.63, 1.05)	0.111		
Smoking history (ref: No)								
Yes	1.42 (1.06, 1.92)	0.020	1.09 (0.72, 1.65)	0.681	1.31 (0.99, 1.73)	0.062	1.21 (0.91, 1.60)	0.201
EGFR mutation (ref: exon 19Del)								
L858R	1.46 (1.09, 1.96)	0.011	1.32 (0.98, 1.77)	0.067	1.32 (1.01, 1.74)	0.045	1.25 (0.95, 1.65)	0.111
Other	1.02 (0.65, 1.60)	0.921	0.95 (0.60, 1.50)	0.812	1.29 (0.86, 1.93)	0.217	1.18 (0.79, 1.77)	0.426
TNM stage (ref: II/III)								
IV	2.15 (0.96, 4.86)	0.064	1.92 (0.84, 4.39)	0.123	1.67 (0.83, 3.39)	0.154		
Brain metastasis (ref: No)								
Yes	1.08 (0.80, 1.45)	0.630			1.08 (0.82, 1.42)	0.602		
Pathology (ref: LUAD)								
LUSC	1.28 (0.60, 2.72)	0.521		1.56 (0.80, 3.05)	0.191			
Other	0.68 (0.17, 2.72)	0.581		0.83 (0.26, 2.58)	0.742			
TKI (ref: Ecetinib)								
Gefitinib	1.16 (0.84, 1.61)	0.363		1.26 (0.93, 1.71)	0.133			
Erlotinib	0.89 (0.57, 1.36)	0.578		0.85 (0.57, 1.28)	0.446			
Other	1.51 (0.37, 6.22)	0.566		1.05 (0.26, 4.30)	0.947			
Surgery (ref: No)								
Yes	0.55 (0.39, 0.78)	0.001	0.60 (0.42, 0.86)	0.005	0.73 (0.53, 1.00)	0.050	0.75 (0.54, 1.03)	0.074

*CI* Confidence interval, *EGFR* Epidermal growth factor receptor, *HR* Hazard ratio, *LUAD* Lung adenocarcinoma, *LUSC* Lung squamous cell carcinoma, *TKI* Tyrosine kinase inhibitor, *TNM* Tumor node metastasis

### Subgroup analysis of risk score in patients with NSCLC receiving EGFR-TKI

High-risk score was associated with poor prognosis in all types of NSCLC patients. To evaluate which subgroups of patients have a predictive effect on prognosis, subgroup analysis was further performed. The risk score was a prognostic biomarker for predicting both OS and PFS in NSCLC patients with *EGFR exon19Del* mutation, *EGFR L858R* mutation, TNM stage IV, LUAD, and treatment with gefitinib, and predicting PFS in patients with young (< 60 y), male, and not receiving surgery treatment (Figures S8–S10).

### Nomogram model visually for predicting OS rate in NSCLC patients

**Fig. 3:**
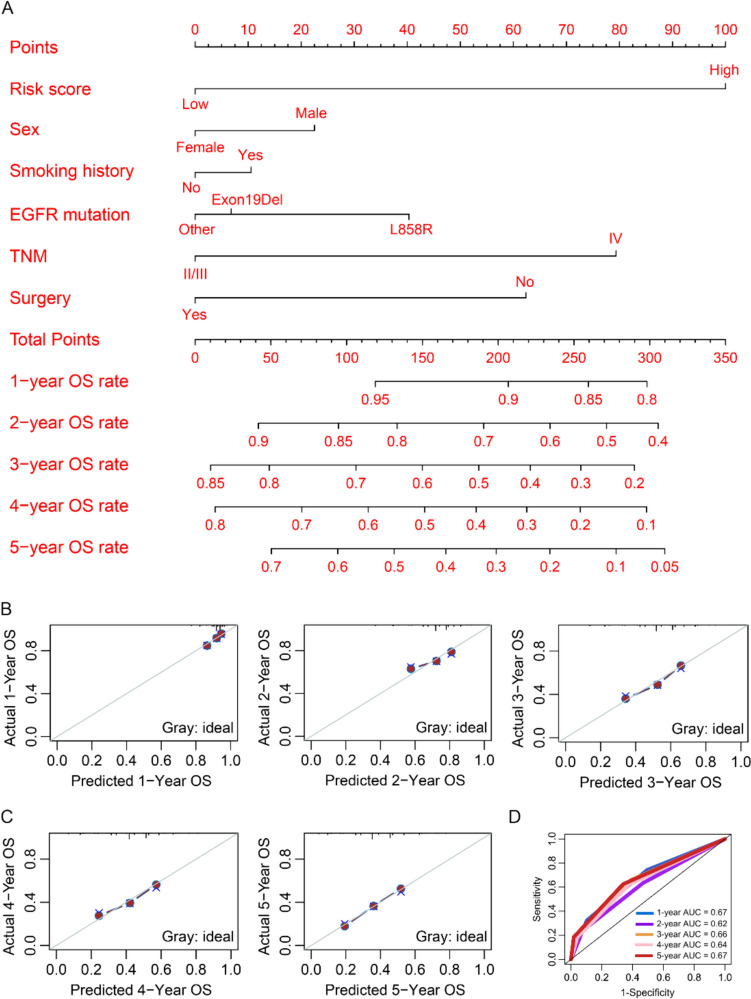
Construction of nomogram model. **A** The risk score, sex, smoking history, *EGFR* mutation, TNM stage, and surgery treatment were used to construct the nomogram model. **B**–**C**: The consistency between the predicted 1-, 2-, 3-, 4-, and 5-year OS rates of the nomogram model and the actual OS rates was validated using calibration curves. **D** Time-dependent ROC curve was used to evaluate the performance of the nomogram model in predicting 1-, 2-, 3-, 4-, and 5-year OS rates

To construct a nomogram model, variables significantly correlated with OS were first selected in the Cox regression analysis. The risk score, sex, smoking history, *EGFR* mutation, TNM stage, and surgery treatment were significantly associated with OS in NSCLC patients receiving EGFR-TKI (Table 1), which were included in the construction of nomogram model for visually and personally predicting 1-, 2-, 3-, 4-, and 5-year OS rates (Fig. 3A). Finally, the calibration curves and time-dependent ROC curves were used to evaluate the performance of the nomogram model in predicting prognosis, and the results suggested that the predicted 1-, 2-, 3-, 4-, and 5-year OS rates of the nomogram model were highly consistent with the actual OS rates, and the AUC was greater than 0.6 (Fig. 3B–D). Taken together, the nomogram model has good performance in predicting 1-, 2-, 3-, 4-, and 5-year OS rates in NSCLC patients.

### Risk stratification for patients with NSCLC receiving EGFR-TKI

**Fig. 4:**
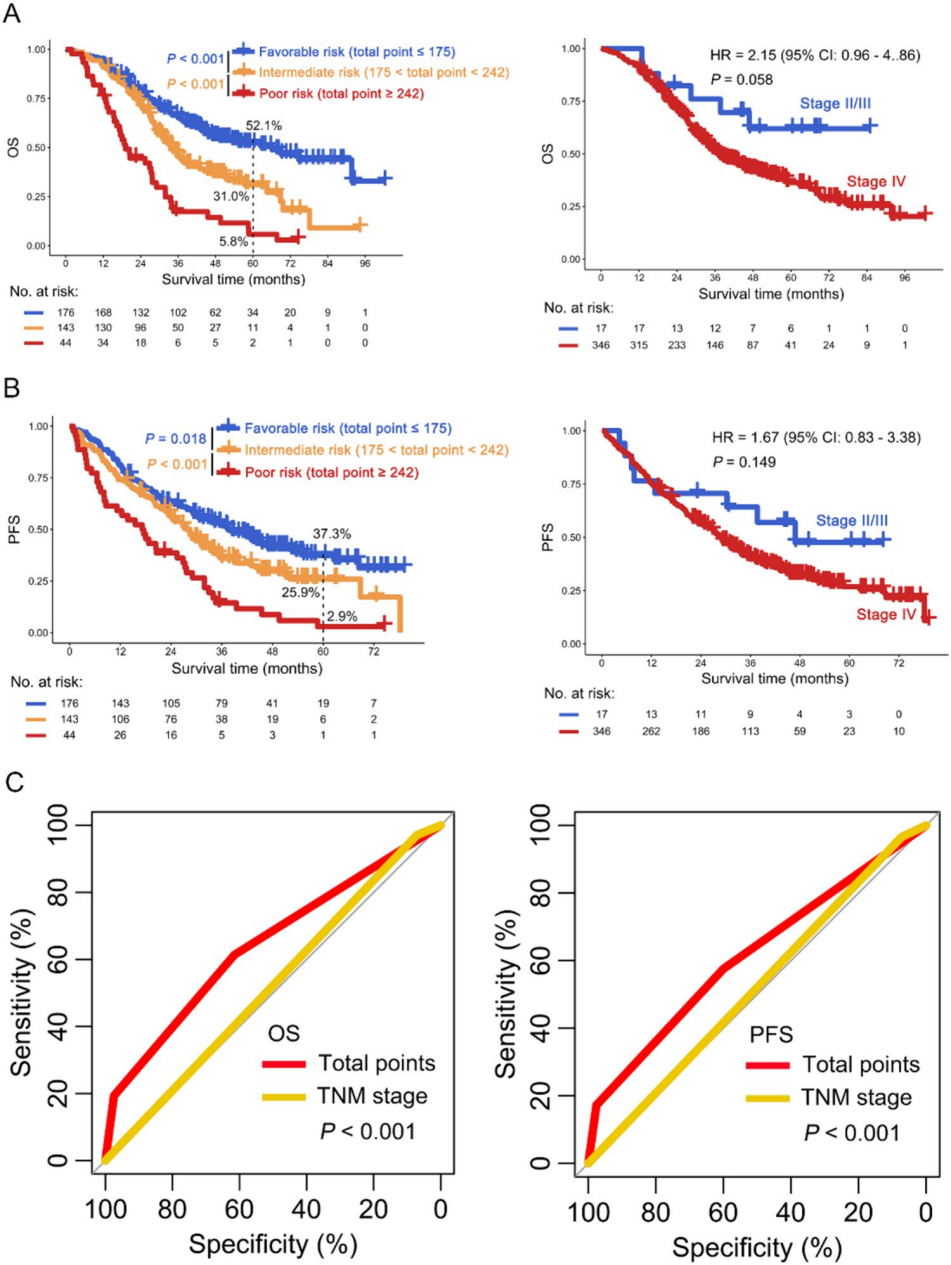
Construction of risk stratification for NSCLC patients treated with EGFR-TKI. A-B: Kaplan–Meier curves of new risk stratification based on nomogram model (left panel) and standard risk stratification based on TNM stage (right panel) in OS (**A**) and PFS (**B**). **C** ROC curves were used for evaluating the performance of new and standard risk stratification in OS (left panel) and PFS (right panel)

Risk stratification provides an important reference for clinicians to manage cancer patients and determine treatment options. Given this, we attempt to construct a risk stratification according to total points derived from the nomogram model for NSCLC patients receiving EGFR-TKI treatment. NSCLC patients were divided into favorable-, intermediate-, and poor-risk subgroups for OS based on the optimal cut-points of total points (175 and 242) (poor vs. intermediate vs. favorable risk: 5-year OS 5.8% vs. 31.0% vs. 52.1%; *P* < 0.001) (Fig. 4A, left panel and S11). However, the TNM stage cannot perform risk stratification for NSCLC patients receiving EGFR-TKI treatment (*P* = 0.058, Fig. 4A, right panel). These findings were confirmed in PFS (total point: poor vs. intermediate vs. favorable risk: 5-year PFS 2.9% vs. 25.9% vs. 37.3%, *P* < 0.05; TNM stage: *P* = 0.149) (Fig. 4B). Finally, the ROC curves were used to compare the differences in OS and PFS between the two risk stratification methods mentioned above, and the results indicated that the total point based on the nomogram model was better in risk stratification for OS and PFS of NSCLC patients compared to TNM stage (*P* < 0.001, Fig. 4C).

## Discussion

Given that EGFR-TKI has become the standard treatment for advanced NSCLC with EGFR mutations, but the overall prognosis is still poor, there is an urgent need to identify and validate specific prognostic factors for these patients [20]. Early identification of individuals with poor long-term OS and PFS can enable clinicians to stratify them to correct baseline problems and provide more comprehensive treatment [21]. In recent years, the important role of pre-treatment malnutrition and systemic inflammatory response in the survival of cancer patients has received increasing attention [13, 22]. In fact, malnutrition occurs in up to 75% of cancer patients, and approximately 21–45% of NSCLC patients suffer from malnutrition [23]. Given the high incidence of malnutrition, several malnutrition biomarkers have been developed to reflect the nutritional status and long-term prognosis of these patients [24]. Furthermore, systemic inflammatory biomarkers in peripheral blood can be used to predict clinical outcomes in multiple solid tumors, including colorectal cancer, esophageal squamous cell carcinoma, and NSCLC [25]. The impact of different inflammatory biomarkers on the prognosis of cancer patients may be associated with the tumor immune microenvironment. Changes in the tumor immune microenvironment are closely associated with the distribution of inflammatory and immune cells in peripheral blood, including lymphocytes, neutrophils, and monocytes [26]. Infiltrating lymphocytes stimulate the production of proinflammatory factors and cytotoxic cells, thereby inhibiting tumor cell proliferation and migration [27]. Conversely, neutrophils have been shown to have tumor-promoting properties. An increase in neutrophils in peripheral blood leads to the upregulation of tumorigenic and angiogenic factors, e.g., vascular endothelial growth factor (VEGF), nuclear factor kappa B (NF-κB), CXC chemokine ligand 8 (CXCL8), granulocyte colony-stimulating factor (G-CSF), and transforming growth factor-β1 (TGF-β1), which induce a tumor-promoting environment [25]. It has been reported that high neutrophil counts in peripheral blood are significantly associated with poor prognosis in cancer patients receiving chemotherapy, radiotherapy, or immunotherapy [25]. Meanwhile, elevated monocyte counts in peripheral blood have also been proven to correlate with poor long-term survival in multiple cancers. Mechanistically, monocytes differentiate into tumor-associated macrophages, secreting tumor necrosis factor-α (TNF-α), VEGF, and proteases to promote tumor growth, angiogenesis, and degradation of the extracellular matrix, thereby inhibiting anti-tumor immune responses in the body and promote tumor cell migration [28]. Ultimately, these different immune cells collectively constitute the tumor immune microenvironment, which has a significant impact on the efficacy and prognosis of cancer treatments [25].

To combine various biomarkers of malnutrition and inflammation, previous studies have developed several composite systemic inflammatory indexes, including SIRI, ALI, LMR, NLR, PLR, PNI, and SII, and malnutrition indexes, including NRI and CONUT, are significantly correlated with the prognosis of NSCLC patients receiving EGFR-TKI treatment in this study. However, the combination of indexes often predicts the prognosis of patients better than a single one. Interestingly, a combination of NRI and SIRI was the best model for predicting clinical outcomes in NSCLC patients treated with EGFR-TKI, especially as a prognostic biomarker for predicting both OS and PFS in patients with *EGFR exon19Del* mutation, *EGFR L858R* mutation, TNM stage IV, LUAD, and treatment with gefitinib and predicting PFS in patients with young (< 60 y), male, and not receiving surgery treatment. NRI integrates parameters of albumin and body weight, while SIRI integrates parameters of three inflammatory cells (lymphocytes, neutrophils, and monocytes), both of which have been proven to be effective prognostic indicators in various tumor models [25, 29]. In addition, previous studies have demonstrated that high NRI and SIRI values are associated with poor prognosis in NSCLC patients [25]. Accordingly, to our knowledge, the prognostic value of combining NRI and SIRI in NSCLC patients receiving EGFR-TKI treatment was little known. Therefore, an advantage of this study is that it confirms that NRI and SIRI are the optimal combination model for predicting prognosis for NSCLC patients treated with EGFR-TKI, especially NSCLC patients with TNM stage IV.

Based on the tumor infiltration range, number of lymph node metastases, and distant metastasis in TNM staging, NSCLC patients can be divided into stages I, II, III, and IV, which provides important reference for disease management, surveillance for relapse, and subsequent treatment [20]. However, over 40% of patients are diagnosed with TNM stage IV [30]. In this study, up to 95.3% of NSCLC patients receiving EGFR-TKI treatment were diagnosed with stage IV. Therefore, risk stratification based on the TNM stage cannot provide relatively accurate predictions for the prognosis of all NSCLC patients receiving EGFR-TKI treatment. Recently, the nomogram model based on prognostic clinical characteristics has been widely used to predict the prognosis and risk stratification for cancer patients [16, 17]. In this study, risk score calculated by weighted NRI and SIRI, combined with sex, smoking history, *EGFR* mutation, TNM stage, and surgery treatment, were used to construct a nomogram model that can visually and personally predict 1-, 2-, 3-, 4-, and 5-year OS rates for NSCLC patients treated with EGFR-TKI and has a good predictive performance. Importantly, NSCLC patients were divided into three subgroups, including favorable, intermediate, and poor risk, according to the optimal cut-points of total points (175 and 242), which significantly predict the OS and PFS for NSCLC patients. Notably, risk stratification based on the nomogram model was better than that based on the TNM stage. Taken together, the risk score calculated by weighted NRI and SIRI, combined with prognostic clinical characteristics, performed risk stratification better than the TNM stage for NSCLC patients treated with EGFR-TKI.

However, some limitations of this study are worth considering. Firstly, due to the retrospective design, it is not possible to reduce the statistical bias between NRI/SIRI and clinical outcomes. Secondly, this study is a single-center result, and data support from multiple centers is needed in the future. Furthermore, the construction of the nomogram model is based on clinical data from a single center. Although the nomogram model has been internally validated through calibration and time-dependent ROC curves, further research is needed in another clinical center to externally validate the proposed nomogram model. Finally, the number of cases in this study is relatively small, and more cases and multicenter data need to be collected in the future to verify the application value of risk stratification in clinical practice.

## Conclusions

High NRI and SIRI were significantly associated with poor OS and PFS in NSCLC patients, making them the best combination models for predicting the clinical outcomes of patients. Importantly, a nomogram model was constructed by combining NRI/SIRI, sex, smoking history, *EGFR* mutation, TNM stage, and surgery treatment to visually and personally predict the 1-, 2-, 3-, 4-, and 5-year OS of patients with NSCLC. NRI/SIRI may be a novel biomarker used to supplement risk stratification in NSCLC patients receiving EGFR-TKI treatment.

## Supplementary Information

Below is the link to the electronic supplementary material.

Supplementary file1 (DOCX 4006 KB)

## Data Availability

The datasets used and analyzed in the current study are available from the corresponding author upon reasonable request.
