# Supplementary material for: NRI and SIRI are the optimal combinations for prognostic risk stratification in patients with non-small cell lung cancer after EGFR-TKI therapy
Source: Clin Transl Oncol. 2024 Sep 20;27(4):1529–38. doi: 10.1007/s12094-024-03735-7 (PMC12000150; doi:10.1007/s12094-024-03735-7)
Supplement: Supplementary file 1 — Supplementary file1 (DOCX 4006 KB) [file 12094_2024_3735_MOESM1_ESM.docx]

**NRI and SIRI are the optimal combinations for prognostic risk stratification in patients with non-small cell lung cancer after EGFR-TKI therapy**


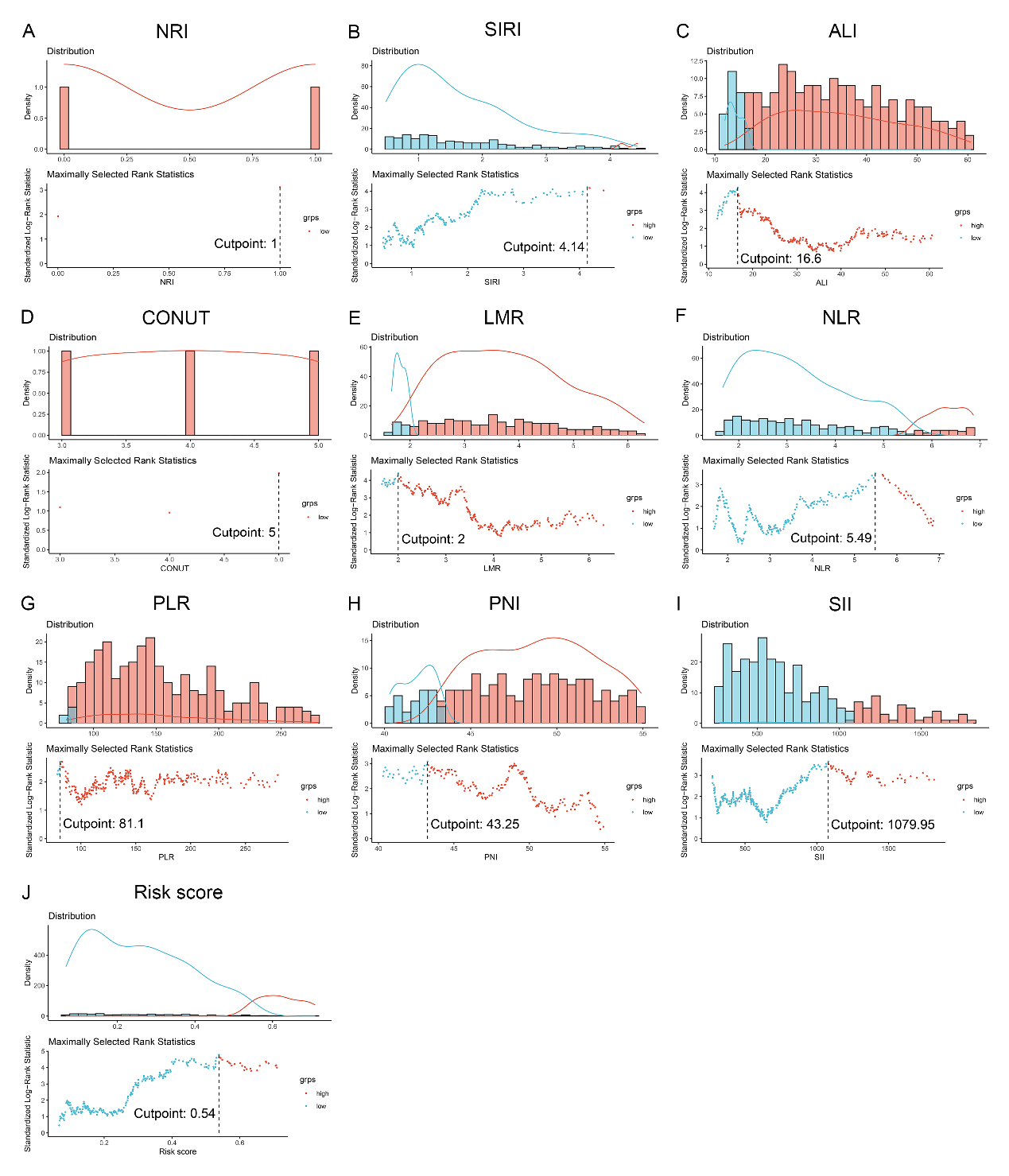


**Figure S1.** A-J: The optimal cut-points for NRI, SIRI, ALI, CONUT, LMR, NLR, PLR, PNI, SII, and risk score in NSCLC patients treated with EGFR-TKI.


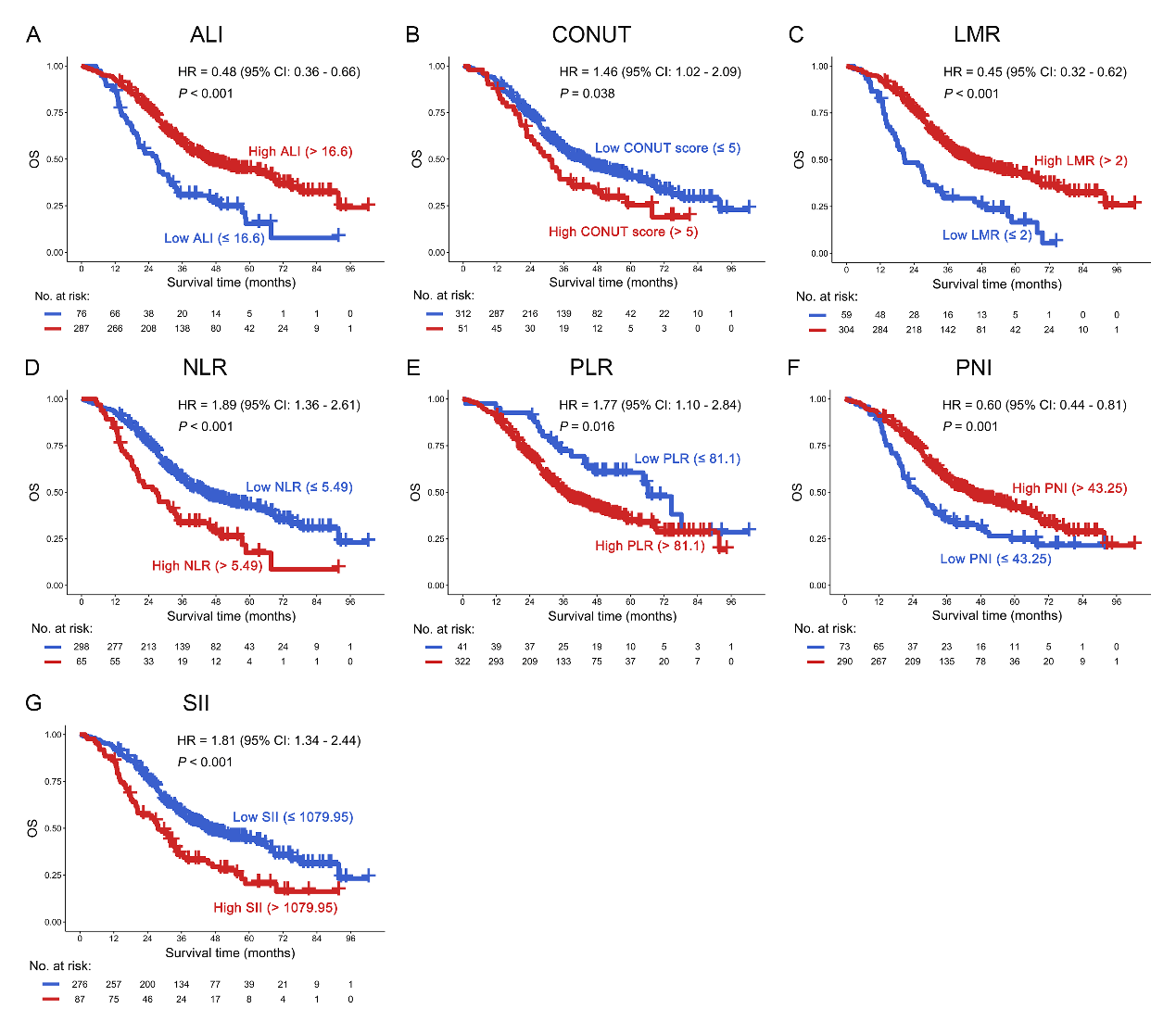


**Figure S2.** OS analysis of ALI (A), CONUT (B), LMR (C), NLR (D), PLR (E), PNI (F), and SII (G) in NSCLC patients treated with EGFR-TKI.


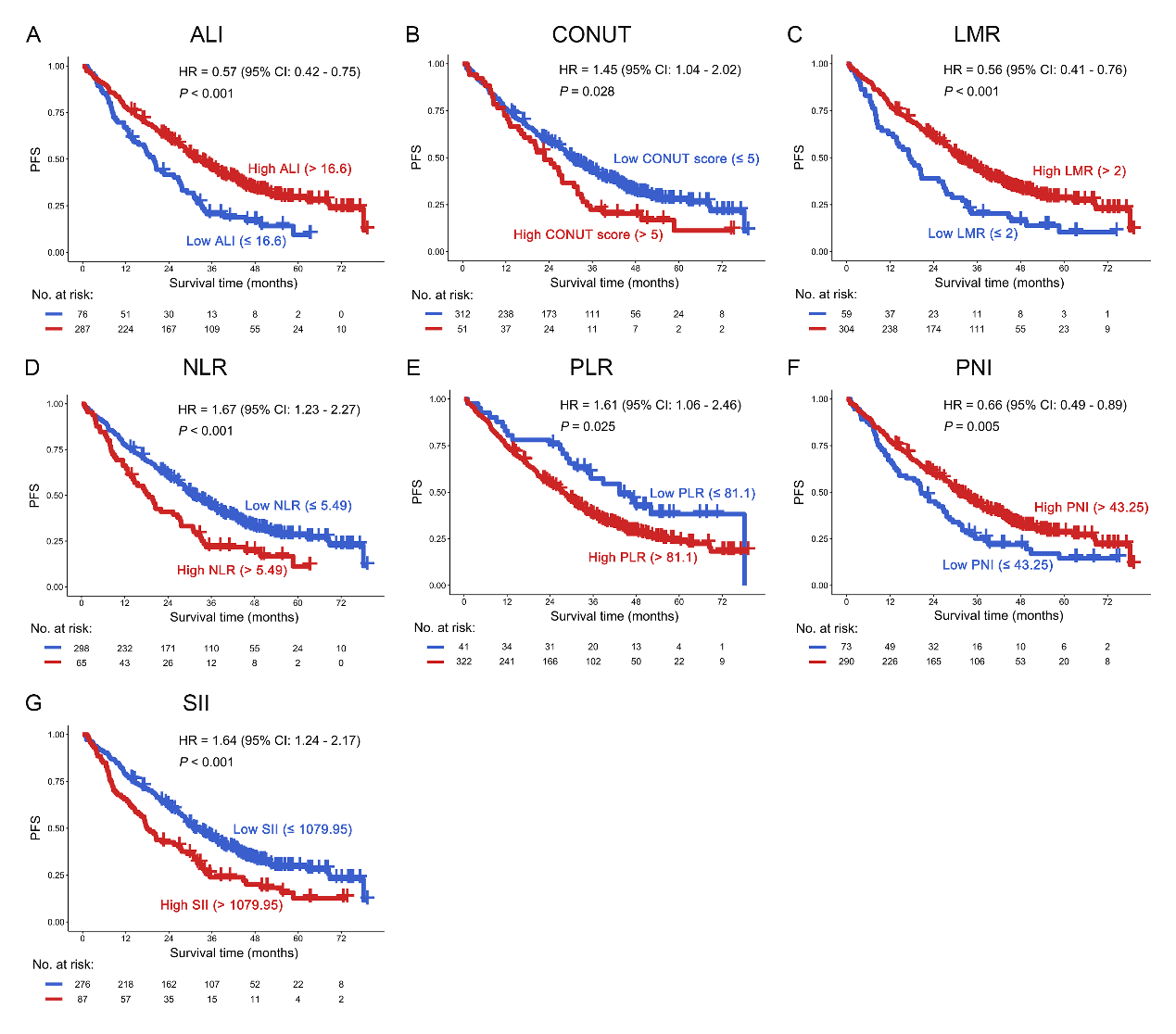


**Figure S3.** PFS analysis of ALI (A), CONUT (B), LMR (C), NLR (D), PLR (E), PNI (F), and SII (G) in NSCLC patients treated with EGFR-TKI.


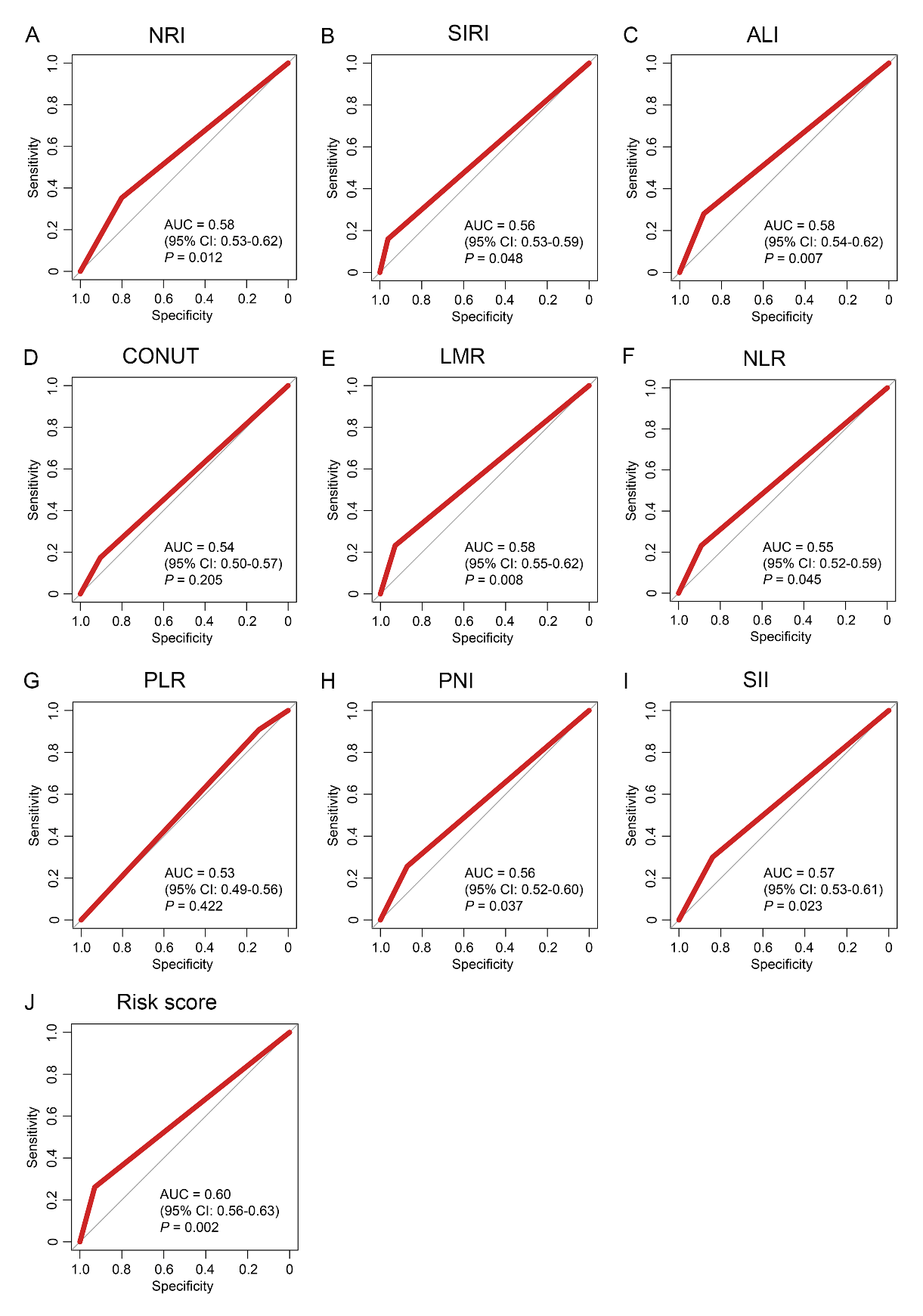


**Figure S4.** The ROC curves of NRI, SIRI, ALI, CONUT, LMR, NLR, PLR, PNI, SII, and risk score for assessing the OS prediction power in NSCLC patients treated with EGFR-TKI.


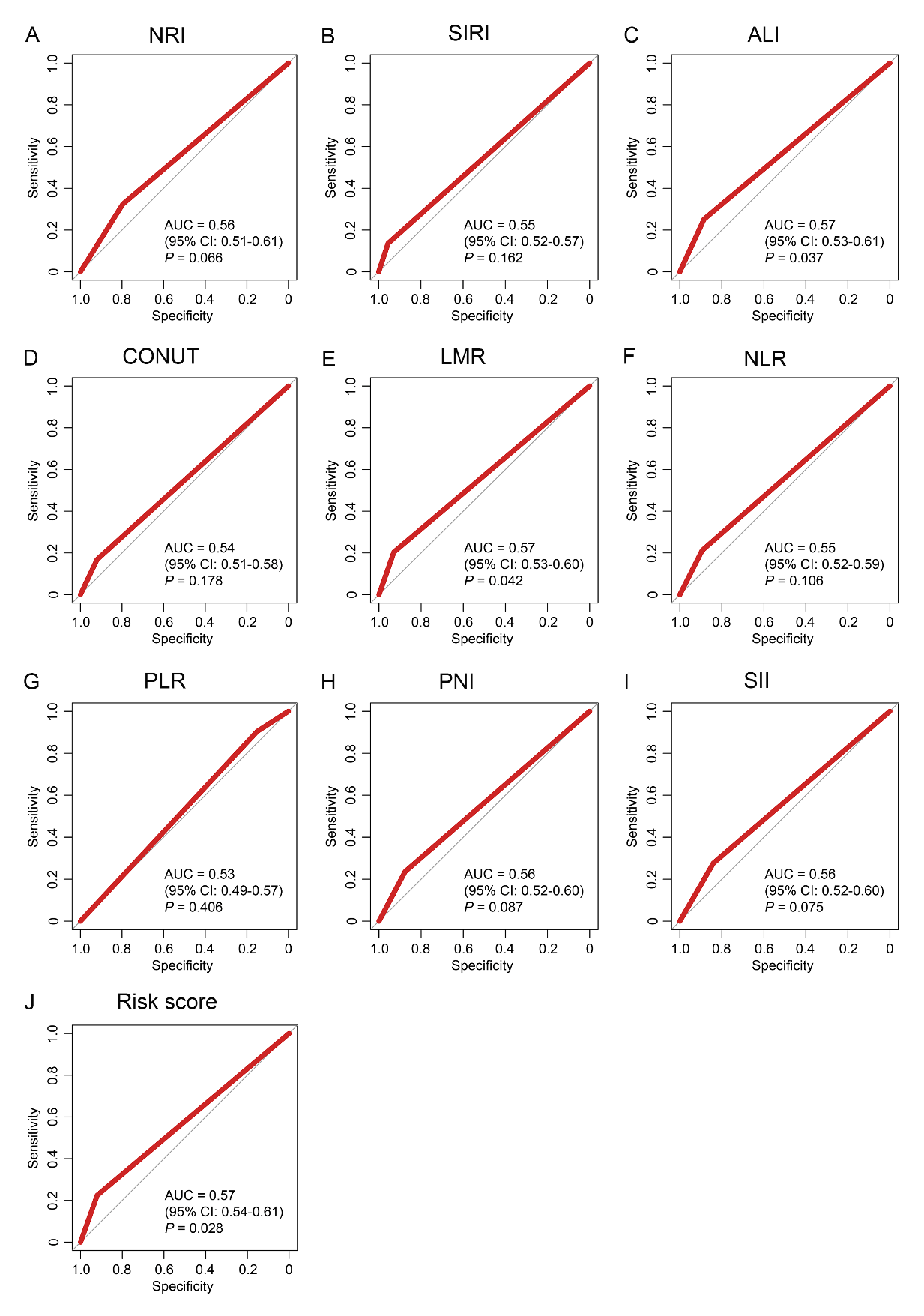


**Figure S5.** The ROC curves of NRI, SIRI, ALI, CONUT, LMR, NLR, PLR, PNI, SII, and risk score for assessing the PFS prediction power in NSCLC patients treated with EGFR-TKI.


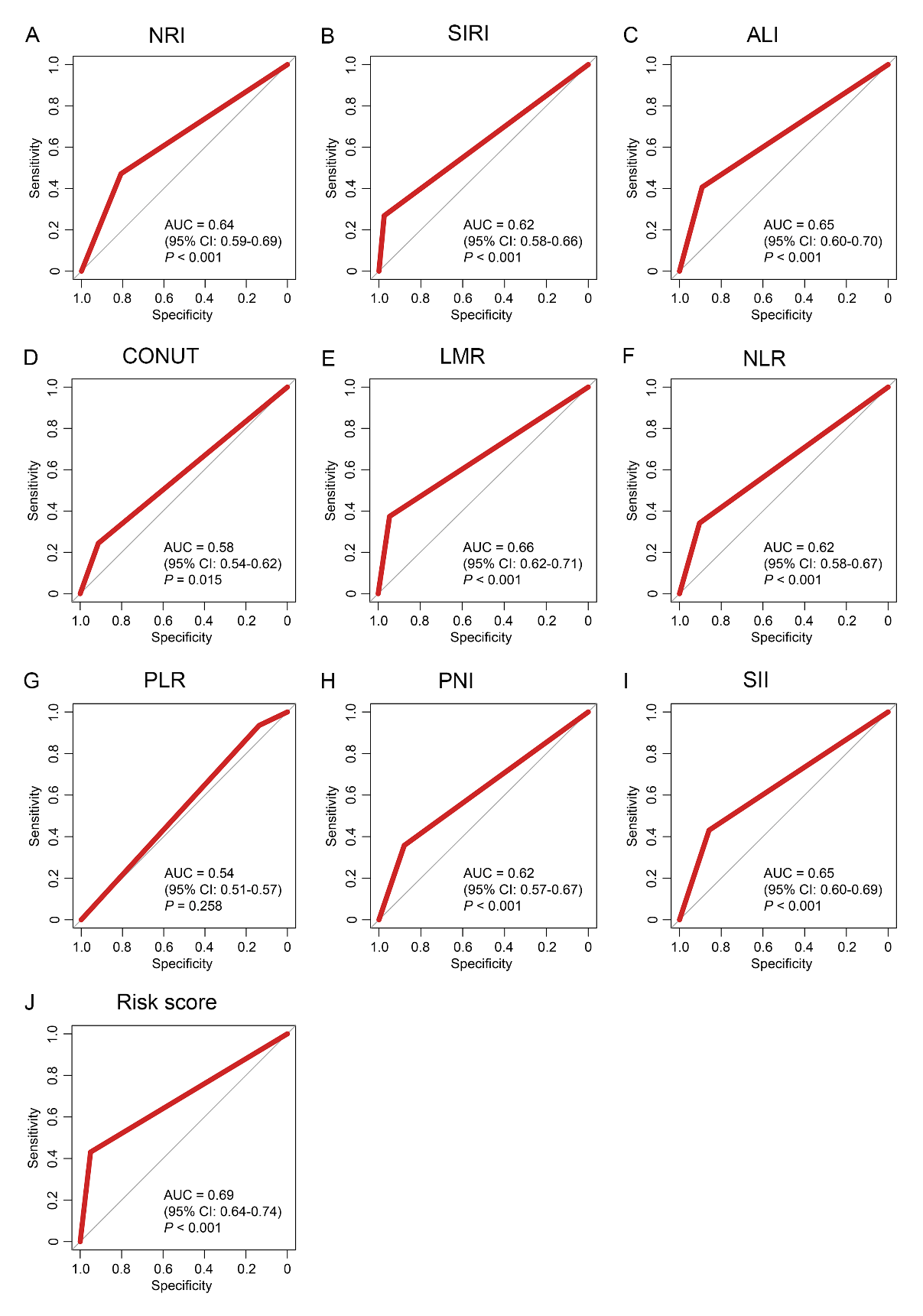


**Figure S6.** The ROC curves of NRI, SIRI, ALI, CONUT, LMR, NLR, PLR, PNI, SII, and risk score for assessing the EGFR-TKI response in NSCLC patients.


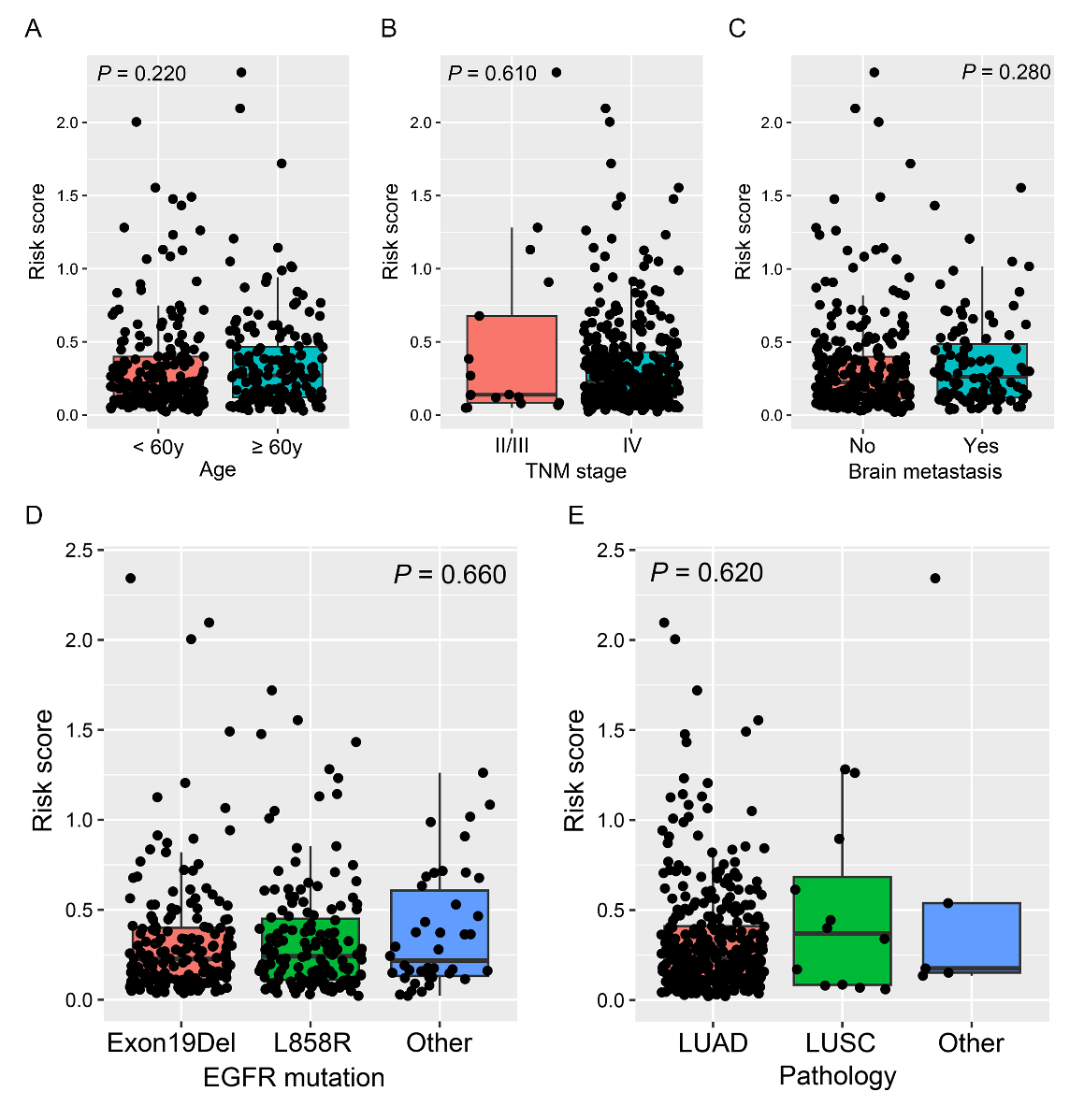


**Figure S7.** The relationship between risk score and age (A), TNM stage (B), brain metastasis (C), *EGFR* mutation (D), and pathology subtypes (E) in NSCLC patients treated with EGFR-TKI.


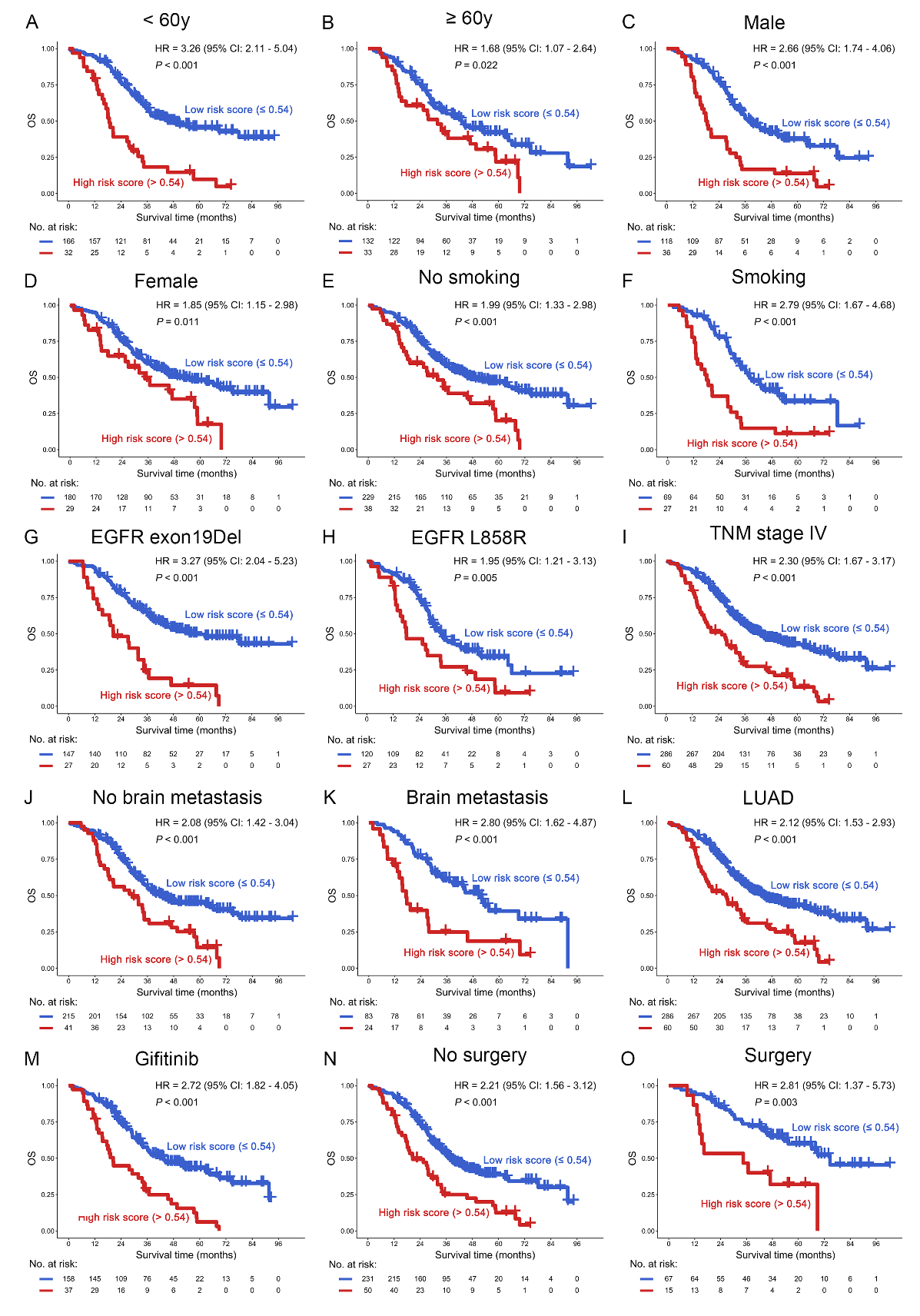


**Figure S8.** OS analysis of risk score in subgroups of patients with NSCLC. A-O: Risk score predicted poor OS for patients with young (<60 y, A), elderly (≥ 60 y, B), male (C), female (D), no-smoking (E), smoking (F), *EGFR exon19Del* mutation (G), *EGFR L858R* mutation (H), TNM stage IV (I), no brain metastasis (J), brain metastasis (K), lung adenocarcinoma (LUAD, L), treatment with gefitinib (M), no surgery (N), or surgery treatment (O).


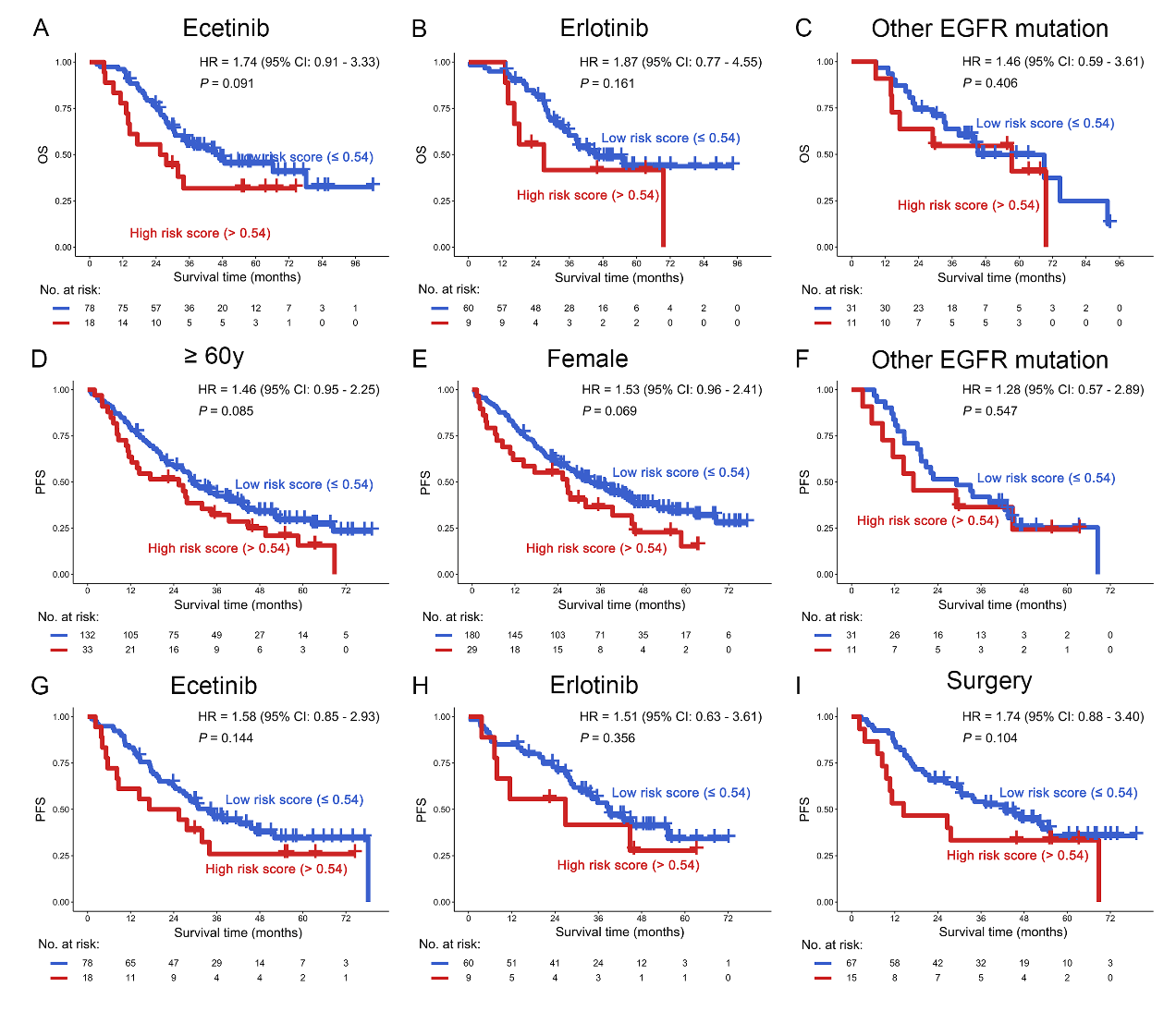


**Figure S9.** OS and PFS analysis of risk score in subgroups of patients with NSCLC. A-C: Risk score cannot predict the OS of patients receiving Ecetinib (A) and Erlotinib (B) treatments, as well as other *EGFR* mutation (C) patients. D-I: Risk score cannot predict the PFS of patients with elderly (D), female (E), other *EGFR* mutation (F), patients receiving Ecetinib (G) and Erlotinib (H) treatments, as well as surgery (I).


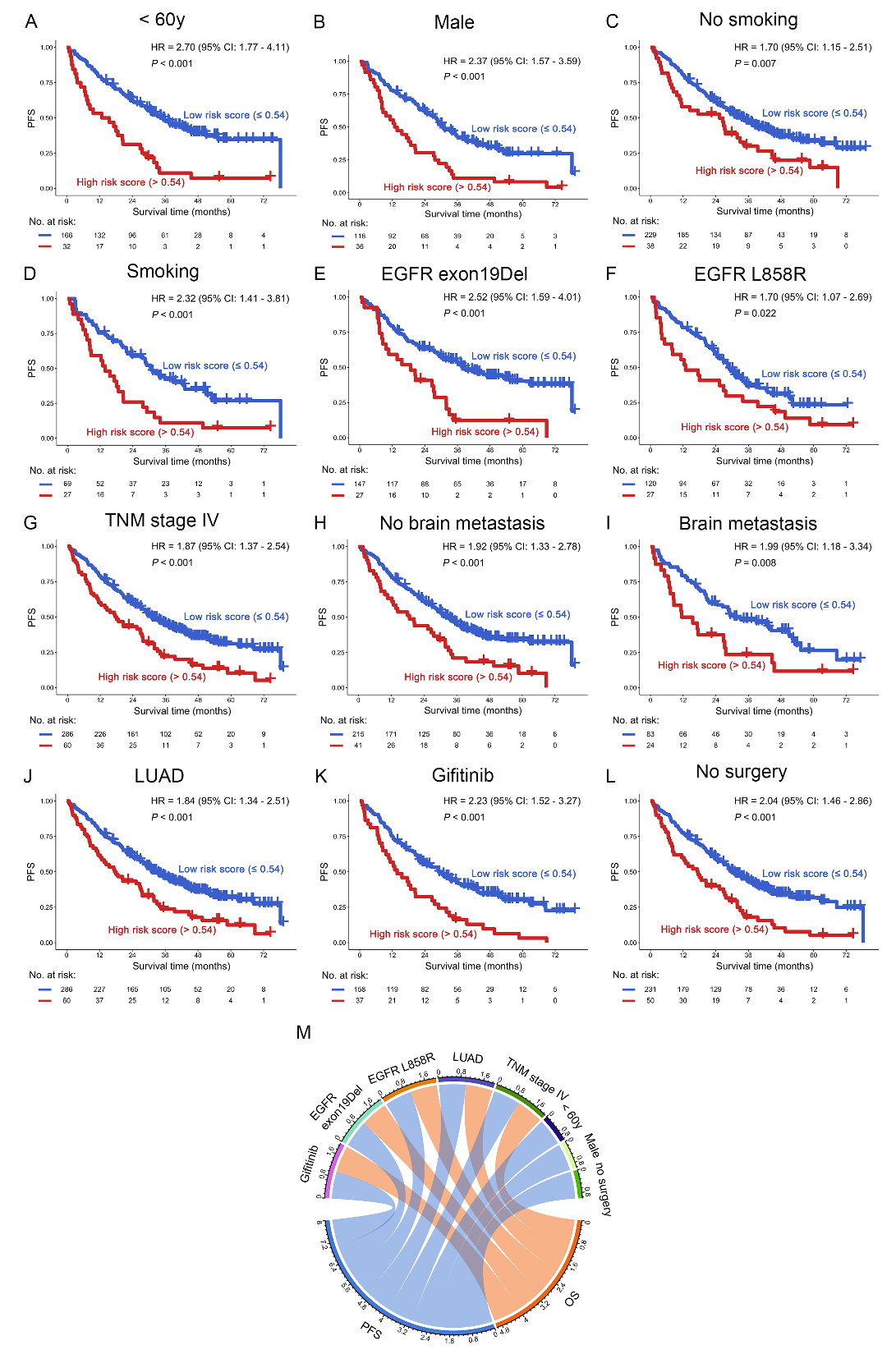


**Figure S10.** PFS analysis of risk score in subgroups of patients with NSCLC. A-L: Risk score predicted poor PFS for patients with young (<60y, A), male (B), no-smoking (C), smoking (D), *EGFR exon19Del* mutation (E), *EGFR L858R* mutation (F), TNM stage IV (G), no brain metastasis (H), brain metastasis (I), LUAD (J), treatment with gefitinib (K), or no surgery treatment (L). (M) Schematic summary of risk score predicting OS and PFS in subgroups of NSCLC patients.


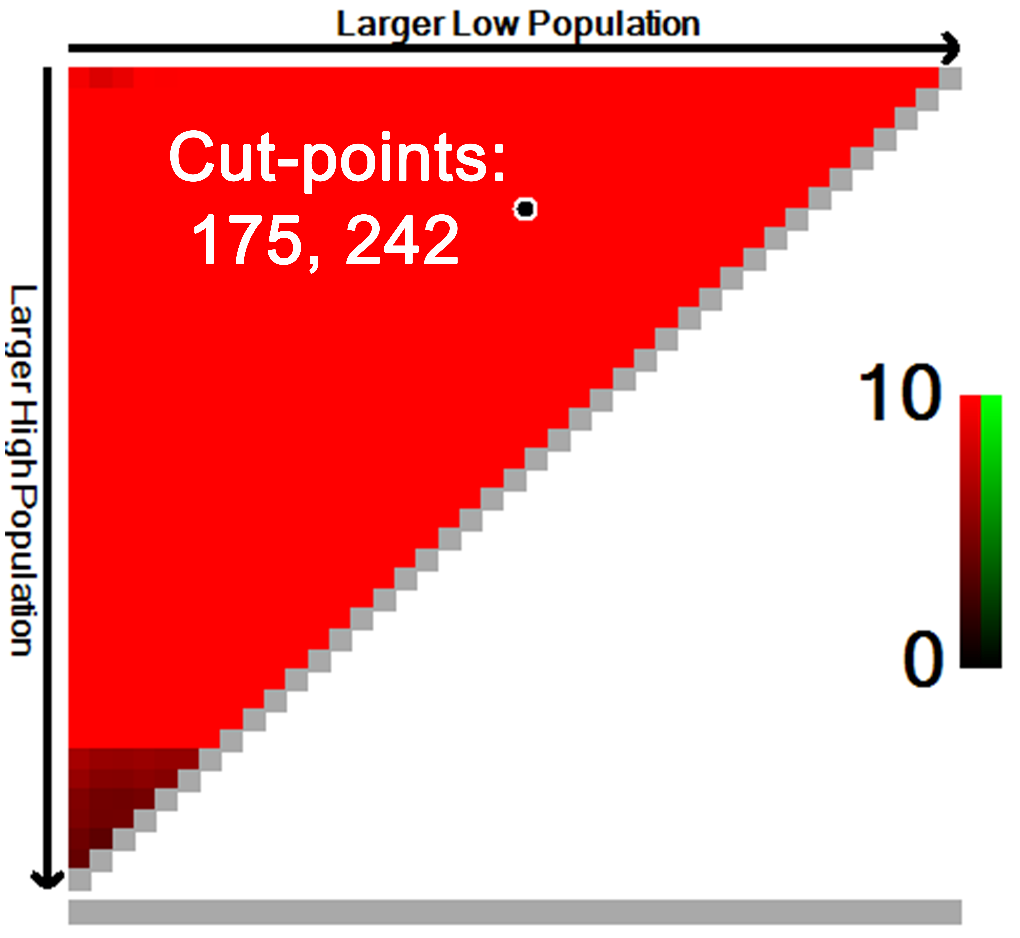


**Figure S11.** X-tile software (version 3.6.1, Yale University, New Haven, CT, USA) was used to determine two optimal cut-points for total point, which divides NSCLC patients into favorable-, intermediate-, and poor-risk subsets. The optimal cut-point appears at the brightest pixel (red or green) of the heatmap.

**Table S1.** Clinical information of patients with lung cancer.

| Variables | N (%) |
| --- | --- |
| Age, years |  |
| < 60 | 198 (54.5) |
| ≥ 60 | 165 (45.5) |
| Gender |  |
| Male | 154 (42.4) |
| Female | 209 (57.6) |
| Smoking history |  |
| No | 267 (73.6) |
| Yes | 96 (26.4) |
| EGFR mutation |  |
| Exon 19Del | 174 (47.9) |
| L858R | 147 (40.5) |
| Other | 42 (11.6) |
| TNM stage |  |
| II | 1 (0.3) |
| III | 16 (4.4) |
| IV | 346 (95.3) |
| Brain metastasis |  |
| No | 256 (70.5) |
| Yes | 107 (29.5) |
| Pathology |  |
| LUAD | 346 (95.3) |
| LUSC | 12 (3.3) |
| Other | 5 (1.4) |
| TKI |  |
| Ecetinib | 96 (26.4) |
| Gefitinib | 195 (53.7) |
| Erlotinib | 69 (19.0) |
| Other | 3 (0.8) |
| Response to TKI |  |
| Yes | 240 (66.1) |
| No | 123 (33.9) |
| Surgery |  |
| No | 281 (77.4) |
| Yes | 82 (22.6) |
| NRI, mean ± SD | 0.68 ± 0.94 |
| SIRI, mean ± SD | 1.97 ± 2.23 |
| ALI, mean ± SD | 34.38 ± 20.44 |
| CONUT, mean ± SD | 4.15 ± 1.42 |
| LMR, mean ± SD | 3.83 ± 2.08 |
| NLR, mean ± SD | 3.88 ± 3.11 |
| PLR, mean ± SD | 167.82 ± 96.13 |
| PNI, mean ± SD | 48.00 ± 5.78 |
| SII, mean ± SD | 899.87 ± 975.42 |

ALI: Advanced lung cancer inflammation index; CONUT: Controlling nutritional status; EGFR: Epidermal growth factor receptor; LMR: Lymphocyte-to-monocyte ratio; LUAD: Lung adenocarcinoma; LUSC: Lung squamous cell carcinoma; NLR: Neutrophil to lymphocyte ratio; NRI: Nutritional risk index; PLR: Platelet-to-lymphocyte ratio; PNI: Prognostic nutritional index; SD: Standard deviation; SII: Systemic immune-inflammation index; SIRI: Systemic inflammation response index; TKI: Tyrosine kinase inhibitor; TNM: Tumor node metastasis.
